# Long-Term Comparison of Survival and Marginal Bone of Implants with and without Sinus Augmentation in Maxillary Molars within the Same Patients: A 5.8- to 22-Year Retrospective Study

**DOI:** 10.3390/jcm10071360

**Published:** 2021-03-25

**Authors:** Won-Bae Park, Ji-Young Han, Kyung-Lhi Kang

**Affiliations:** 1 Department of Periodontology, School of Dentistry, Kyung Hee University, Seoul 05278, Korea; njkysh@naver.com; 2 Department of Periodontology, Division of Dentistry, College of Medicine, Hanyang University, Seoul 04763, Korea; hjyperio@hanyang.ac.kr

**Keywords:** bone resorption, dental implants, sinus floor augmentation, survival rate

## Abstract

Maxillary sinus floor augmentation (MSFA) is widely used and considered a predictable procedure for implant placement. However, the influence of MSFA on implant survival and marginal bone loss (MBL) is still inconclusive. The purpose of this retrospective observational study is to evaluate the long-term genuine influence of MSFA on the survival and MBL of implants by comparing those with and without MSFA only in maxillary molars within the same patients. Thirty-eight patients (28 male and 10 female), with a total of 119 implants, received implants with and without MSFA, and were followed up for 5.8 to 22 years. Patient- and implant-related factors were assessed with a frailty model for implant survival and with generalized estimation equations (GEE) for MBL around the implant. No variables showed a statistical significance for implant failure in the frailty model. In GEE analysis for MBL, MSFA did not show any statistical significance. In conclusion, MSFA demonstrated no significant influence on implant failure and MBL in posterior maxilla in this study.

## 1. Introduction

Since the first introduction of maxillary sinus floor augmentation (MSFA) by Boyne and James [1], MSFA has been a widely used and predictable procedure for implant placement in severely resorbed and/or pneumatized posterior maxilla [2,3,4]. MSFA is a technically difficult procedure that can be achieved by either the crestal approach or the lateral window approach. Implants can be installed simultaneously with the MSFA procedure or following healing of the augmented area. Moreover, many other factors including bone graft material, residual bone height (RBH), intra- or post-operative complications, implant type, and host factors can affect the survival or success of implants placed in MSFA area [5,6,7]. Though calvarial bone could be used successfully for a donor site to get enough autogenous bone as the gold standard of bone graft material, many bone substitutes are replacing autogenous bone in a minimally invasive surgery, especially in compromised patients [8].

Many studies have investigated the survival and success rates, or marginal bone loss (MBL), of implants placed in augmented maxillary sinus and native bone. Some articles reported similar results when comparing implants in the augmented sinus with those in the native bone in the posterior maxilla [9,10], some of which compared implants placed in the MSFA area with even shorter implants in the native bone [11,12,13]. However, other studies have reported negative results of implants placed in the augmented sinus with the same comparison [14,15,16].

Graziani et al. reported a wider range of implant survival in MSFA (36% to 100%) than in implants placed in the pristine posterior maxilla (73% to 100%) at patient level in their systematic review [17]; however, they could not elucidate the reasons for the variability due to the heterogeneity of studies. The effects of multiple variables, including the sinus elevation technique, use and type of graft material, use of a barrier membrane over the lateral window, and timing of implant placement, were studied or mentioned in some systematic reviews about the clinical performance of implants with MSFA [9,17,18]. These factors can hinder a consistent result of studies on those implants. As far as we know, there has not been a study of exclusive comparison implants in MSFA with those in native bone in posterior maxilla by a single dentist within every single patient in order to control host factors and operator factors. Moreover, with regards to the selection of a proper statistical test for dental research, it is critical to determine whether the data were from teeth from one patient or teeth from among different patients, because implants within one patient have a closer correlation to each other than implants in separate patients do [19,20].

Therefore, this study aimed to evaluate the influence of MSFA on the survival and MBL of implants placed simultaneously with MSFA through the lateral window approach compared to those in the native bone only in the posterior maxilla within every single patient who received both surgeries successively within 6 months and had similar systemic conditions.

## 2. Materials and Methods

### 2.1. Study Design

**Figure 1:**
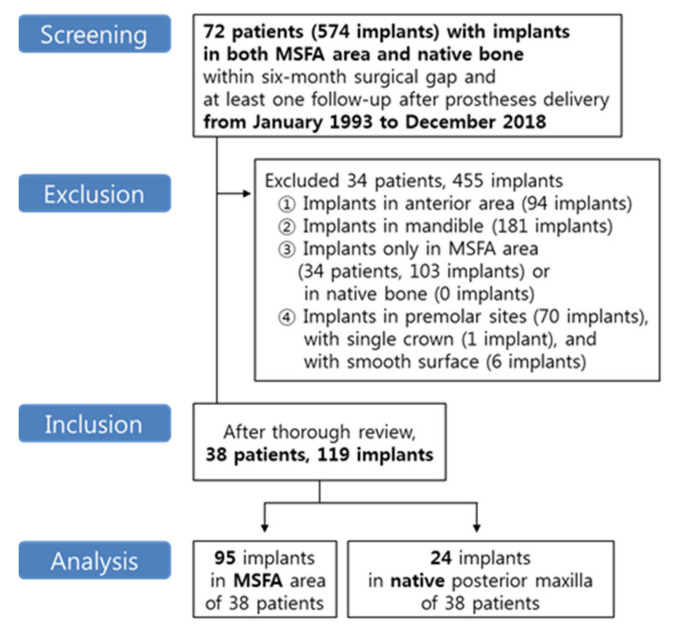
Study flow.

The treatment records of all patients who received implant placements in both: the MSFA area with the simultaneous MSFA procedure; and in the native bone of the posterior maxilla using the conventional technique—with both implants performed between January 1993 and December 2018—were screened. We included patients who were followed up at least once after prostheses delivery to December 2018, and the term between implant placements in augmented bone and native bone was limited to within 6 months in order to control for host factors such as healing potential. Also, other conditions affecting osseointegration or MSFA were considered. To evaluate the genuine influence of MSFA by comparing implants in the MSFA area and the native posterior maxilla, implants in the anterior area and mandible were excluded. Patients with implants in only the MSFA area or only the native posterior maxilla were excluded, too. Finally, implants with a single crown or smooth surface were excluded for homogeneity, and implants in premolar sites were also excluded to compare only molar implants with and without MSFA. This study flow is shown in Figure 1. All surgeries and prosthetic procedures were conducted at a private dental clinic by one experienced clinician. This study was approved by the Korea National Institute for Bioethics Policy (PO1-201808-21-014). Strengthening the Reporting of Observational Studies in Epidemiology (STROBE) guidelines were followed for preparing this manuscript.

#### 2.1.1. Inclusion Criteria

1. Patients who had both simultaneous implant placements with MSFA through the lateral window approach and conventional implant placement in the native bone in their oral cavities;
2. The term between the two types of implant placement was less than six months;
3. Patients who were followed up at least once after prostheses delivery.

#### 2.1.2. Exclusion Criteria

1. Patients who were less than 19 years old at the time of implant surgery;
2. Patients who had any systemic diseases or medications that affect bone metabolism or wound healing at or before the time of implant surgery;
3. Patients who had a history of head or neck radiation therapy at or before the time of implant surgery;
4. Patients who had signs or symptoms of maxillary sinusitis or a history of maxillary sinus surgery at or before the time of implant surgery;
5. Patients who had implants placed in the anterior area, the mandible, only the MSFA area, or only the native posterior maxilla;
6. Implants placed in premolar sites or implants with very low frequency in one group (for example, one implant with single crown and six implants with smooth surface in native bone).

### 2.2. Treatment Records Review

From a chart review, we gathered the following data: demographic data (age, sex, smoking habit, rhinitis, diabetes, and osteoporosis at the time of implant placement); date of surgery (implant placement with or without MSFA), prostheses delivery, last follow-up visit, and implant removal where applicable; time of implant placement after extraction (three groups: immediately after extraction, 2–4 months after extraction, and more than 4 months after extraction); healing period from implant placement to prostheses delivery; follow-up period from prostheses delivery to the last visit or removal; location of implant; implant length and surface characteristics (two groups: smooth or rough); RBH; perforation of the sinus membrane during MSFA; maxillary sinusitis after MSFA; and implant survival. Implant failure was defined as the removal of an implant from the alveolar ridge for any reason. Survival time (i.e., follow-up time) was defined as the time from the delivery of the implant prostheses to the implant removal or the last follow-up.

### 2.3. Surgical Methods

One experienced clinician (W.-B.P.) performed all surgical procedures. Under local anesthesia in the posterior maxilla with lidocaine (1:100,000 epinephrine), the lateral bony window was prepared on the lateral maxillary sinus wall with a low-speed round bur and copious saline cooling after crestal and vertical incision and flap reflection. After elevating the sinus membrane with the attached lateral window bone, a bone substitute (BioOss^®^, Geistlich Pharma AG, Wolhusen, Switzerland) was grafted under the elevated sinus membrane. When the perforation of the sinus membrane was detected, it was carefully elevated and covered with a bovine collagen membrane (CollaTape^®^, Zimmer Biomet, Carlsbad, CA, USA) before bone grafting. Immediately after the implant installation in the MSFA area, more bone substitute was grafted to the level of the lateral window if space around the implants was detected, and primary closure was performed. There was no horizontal or vertical bone augmentation on the ridge. For implant placement without MSFA, conventional procedures like drilling alveolar bone, implant installing, and suturing flaps were performed after local anesthesia in the posterior maxilla. The patients took antibiotics and analgesics for seven days, and rinsed their mouth with 0.12% chlorhexidine (Hexamedine^®^, Bukwang Pharm., Seoul, Korea) at least twice a day for two weeks because chlorhexidine was known as the gold standard of antimicrobial agents until recently [21,22]. Approximately 3 to 20 months following implant placement, the final prostheses were delivered. Follow-up visits were scheduled every six months thereafter for plaque control and periodic check-up. The patients were asked to visit the dentist when they felt pain or some problems.

### 2.4. Radiographic Examination

All patients received panoramic radiographs before surgery, after implant placement with or without MSFA, after prosthetic restoration, and at follow-up visits. Immediately after prosthesis delivery and at follow-up visits, intraoral periapical views were taken. Two blinded examiners (J.-Y.H. and K.L.K.) measured the RBH and MBL with the Analysis Toolkit (Adobe Photoshop CS6, Adobe Systems Inc., San Jose, CA, USA) and imported panoramic and periapical views.

The RBH before MSFA and MBL was measured using the method described in our previous study [23]. Briefly, for RBH, a panoramic view was taken with a surgical stent, which had a metal tube with an already known length. The magnification errors of the panoramic views could be compensated with the calculation using tube length (i.e., RBH = the measurement of available ridge height/the measured tube length × the known actual tube length). The mean value of the calculated RBHs by two persons was used in the analysis. For MBL, the distance from the implant abutment junction to the most apical bone-to-implant contact was measured at the mesial and distal aspects of the implant in a parallel periapical radiograph after prostheses delivery and at the latest follow-up visits. Then, the actual MBL was calculated with the known length of implant—i.e., MBL = the sum of measured mesial and distal distances/(2 × the measured implant length) × the known actual implant length. The mean value of the MBLs calculated by two people was used as the MBL of the implant.

### 2.5. Statistical Analysis

All data were analyzed with commercially available software programs, R software packages (R version 3.5.1, last uploaded on 2 July 2018, www.r-project.org) and SPSS software version 24.0 (SPSS Inc., Chicago, IL, USA). To compare the survival rate and MBL of the implants placed in MSFA with those in the native bone in the posterior maxilla within the same patient with a constant systemic condition, we screened patients who had implants placed in both sites in the posterior maxilla. Thus, we used the frailty model using the Cox proportional hazards model with mixed effects (R software packages) for the survival analysis of implants, and generalized estimation equation (GEE) analysis (SPSS) for evaluating the factors affecting MBL, since there were at least two multiple implants placed in the posterior maxilla in a single patient. The intraclass correlation coefficient was used to assess the intra-examiner reliability for the measurements of RBH and MBL. *p*-values < 0.05 were considered statistically significant in all analyses.

The implant survival was evaluated by univariable analysis using a frailty model according to age, sex, MSFA, diabetes, osteoporosis, rhinitis, smoking habit, time of implant placement after extraction, implant length and diameter, healing period from implant placement to prostheses delivery, RBH, perforation of sinus membrane during MSFA, maxillary sinusitis after MSFA, and the implant location. Multivariable analysis was performed by adjusting patient-related variables like age, sex, diabetes, osteoporosis, rhinitis, and smoking habits. The hazard ratio (HR) showed the association between each variable and implant failure. A HR equal to 1 would indicate that the groups have the same hazards, while HR < 1 and HR > 1 would indicate less and more risk respectively.

The relationship between MBL and each variable was assessed with a univariable analysis using GEEs for the same variables used in the frailty model for the implant survival analysis [20,24,25]. Then, a multivariable analysis was performed with adjusting patient-related variables. The MBL values were prepared as binary outcome variables with the reference of the median value. A Wald Chi-square test was used to analyze the statistical significance of each parameter within the model. The results of the final model were presented as an estimated odds ratio (OR) for each variable (*p* < 0.05).

Because this is a long-term follow-up study, we analyzed the data based on the follow-up period. Survival and MBL of implants were analyzed with three groups (group 1, 5 ≤ years < 10; group 2, 10 ≤ years < 15; group 3, 15 ≤ years) using simple GEE and multiple GEE models. The results were shown as an OR for survival and estimate for MBL (*p* < 0.05).

## 3. Results

This retrospective observational study consisted of 38 patients (28 male and 10 female), with a total of 119 implants, with a 5.8- to 22-year follow-up. The patients received implant placement simultaneously with MSFA, as well as in the native bone in the maxillary molars from January 1993, and were followed up at least once after prostheses delivery to December 2018. Table 1 shows the basic information of the patients and implants of this study. The mean follow-up period was 12.62 ± 3.64 years at implant level. The age of the patients ranged from 35 to 62 years (50.61 ± 6.83 years) at the time of implant placement. The number of patients who had smoking habits, rhinitis, diabetes, and osteoporosis were 21, 3, 11, and 5 out of 38, respectively. Out of 119 implants, 95 implants were placed simultaneously with MSFA, and 24 implants were placed in the native bone of posterior maxilla. Nine patients and 32 implants (23 implants with MSFA and 9 implants without MSFA) were followed up for 5 to 10 years; 17 patients and 54 implants (41 implants with MSFA and 13 implants without MSFA) for 10 to less than 15 years; and 12 patients and 33 implants (31 implants with MSFA and 2 implants without MSFA) for 15 years and more. The total survival rate of implants in the maxillary molars was 89.08%, while the survival rates in the MSFA and native groups were 89.47% (85 out of 95 implants) and 87.5% (21 out of 24 implants) respectively. The earliest removal of an implant occurred 5.85 years after its placement, and the patient had shown a maxillary sinusitis after MSFA. Besides this, all implants analyzed in this study were the external type, and had a rough surface and splinted-bridge-type prosthesis.

**Figure 2:**
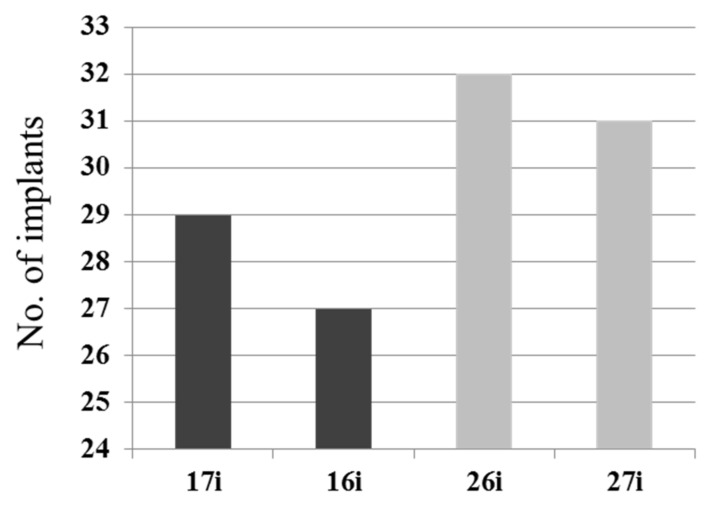
Distribution of molar implants. For the first and second molar replacements, 59 implants (49.6%) and 60 implants (50.4%) were placed, respectively.

Figure 2 shows the location of the implants placed in maxillary molars. For the first and second molar replacement, 59 implants (49.6%) and 60 implants (50.4%) were placed respectively.

The intraclass correlation coefficients for the measured values of RBH and MBL were 0.995 and 0.954, respectively. Distribution of implant length, diameter, and manufacturer is shown in Supplementary Table S1.

Table 2 shows the result of univariable analysis for factors related to implant failure with the frailty model using the Cox proportional hazards model with mixed effects. No variable was statistically significant for implant failure.

In the multivariable analysis for factors related to implant failure, no variables showed statistical significance at implant level (Table 3).

**Figure 3:**
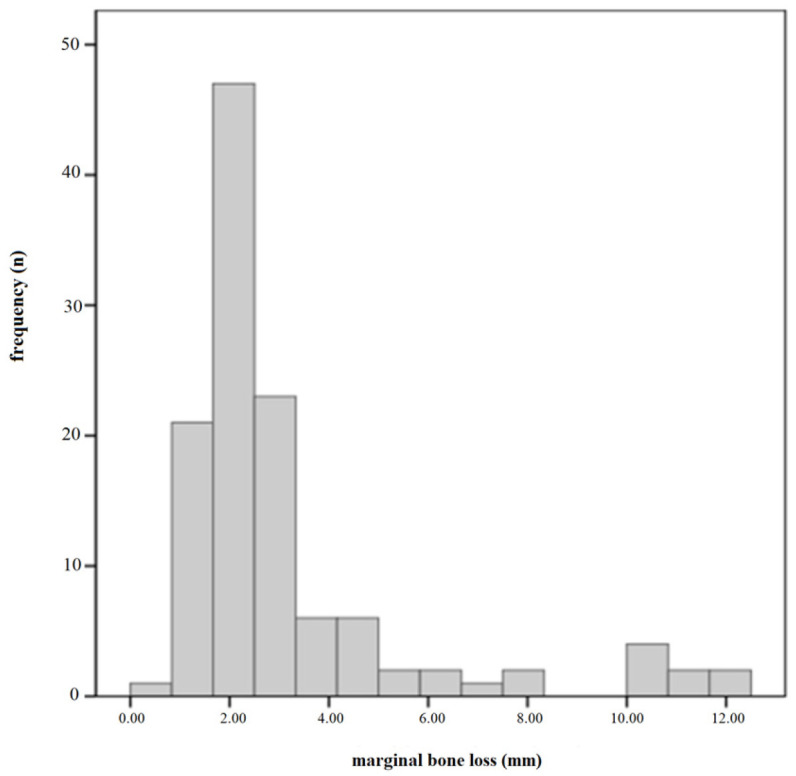
Distribution of marginal bone loss (MBL) at the implant level. The mean MBL (±standard deviation) was 3.15 (±2.51) mm (95% confidence interval: 2.6962–3.6084), and the median value was 2.40 mm.

Figure 3 shows the distribution of MBL at implant level. The mean MBL (±standard deviation) was 3.15(±2.51) mm (95% CI: 2.6962–3.6084), and the median value was 2.4 mm.

Table 4 shows the result of a univariable analysis using GEEs to establish the relationship between MBL and each variable. Table 5 shows the result of multivariable GEE analysis for MBL. The implant location of the second molar site shows about two-fold higher OR compared to that of the first molar site (95% CI: 1.080–3.571, *p* = 0.0270).

Table 6 and Table 7 showed the comparison of implant survival and MBL when the follow-up period was divided into three groups (group 1, 5 ≤ years < 10; group 2, 10 ≤ years < 15; group 3, 15 ≤ years) using simple and multiple GEE models. After adjusting patient-related variables for the multiple GEE model, the OR for implant survival of group 3 increased more than 13 times compared to that of group 1 (Table 6). The estimates for MBL of group 2 and 3 were almost −1.84 and −2.32 compared to that of group 1, respectively (Table 7).

## 4. Discussion

This retrospective study of 38 patients with 119 implants investigated the long-term effect of MSFA through the lateral window approach simultaneous with implant placement on the survival and MBL of implants placed in the maxillary molars. In order to maximize the control of many possible factors affecting the survival and MBL of each implant, implants placed in simultaneous MSFA were compared with those placed in only the native posterior maxilla and within a six-month gap between the two surgeries within a single patient. All surgical and prosthetic procedures were performed by one experienced dentist. Therefore, consistent surgical and prosthetic techniques were maintained and technical differences by heterogenous operators could be excluded. The results showed that the failure risk of implants placed simultaneously with MSFA was not significantly different from that of those placed in the native bone without MSFA after a mean 12.62-year follow up. For MBL ≥ 2.4 mm, the median value of measured MBL values in this study, MSFA showed no statistically significant OR, but the implant location of the second molar showed statistical significance. The risk of MBL ≥ 2.4 mm for the second molar sites was about two times greater than that for the first molar site.

The uniqueness of the present study lies in the limited inclusion and exclusion criteria and statistical analyses; the frailty model using the Cox proportional hazards model with mixed effects for implant survival; and GEE analysis for MBL. In order to control the host factors, only patients with implants placed in simultaneous MSFA, as well as those placed in the native maxillary molars with a time gap between the two surgeries < six months were selected. Moreover, it was possible to control the operator factors since all of the surgical and prosthetic treatments were performed by one dentist. In addition, implants placed in premolar sites or implants with very low frequency only in one group (one implant with single crown and six implants with smooth surface in native bone) were excluded to improve group homogeneity. Thus, implants in this study had rough surfaces and splinted-bridge type prostheses. There was no implant failure before the 5.85-year follow-up. Statistical analysis methods were chosen by considering repeated observations measured in a single patient. Most of the other studies gathered information of many implants from a number of different patients, or from the upper and lower jaws, and studied several variables at implant level. However, this study only included patients with at least two or more implants and in both groups (i.e., MSFA and native posterior maxilla groups), and compared the two groups of implant within each patient. Since the observations measured from implants placed in different patients are not correlated, while those from implants within a single patient are correlated, the data construction based on the correlation has a great effect on the *p*-value in statistical analysis [19,20]. To the best of our knowledge, no previous study has used these analytic methods and inclusion and exclusion criteria to evaluate the genuine effect of MSFA on implant survival and MBL with more than a five-year follow-up.

In recent years, there has been controversy regarding the effect of MSFA on implant survival. With regards to the survival rate of implants placed in the native bone and placed after sinus lift in the posterior maxilla, most studies either reported no significant differences or similar results [9,10,26,27,28,29]. Only a few studies reported greater survival of implants in the native bone than in the MSFA area in the posterior maxilla [14,30,31]. The present study was consistent with the majority of the previous studies, which revealed that the failure risk of implants placed with simultaneous MSFA was not significantly different from that of those in the native posterior maxilla. In this study, a single dentist performed all procedures, and he used one surgical method as well as one kind of bone substitute during the MSFA procedure. There are many confounding factors (i.e., the presence or type of bone graft, type and location of implants, implant placement with or after MSFA, lateral or crestal approach, grafts with or without platelet-rich plasma) related to MSFA procedures, which were also mentioned in previous systematic reviews [9,17,32]. In addition, MSFA procedures are technically demanding, and the surgical outcome depends on the operator’s proficiency, making clinical implant studies involving MSFA notoriously difficult. Furthermore, the present study included implants placed exclusively in the first and second maxillary molars because of the different magnitude of loading force between the anterior and posterior teeth and different bone quality between the maxilla and mandible. Although this study showed that MSFA was not significantly dangerous for implant survival with limited criteria, it might have a different result with more samples. Therefore, further clinical studies should be performed with more samples and controlling the confounding factors for the appropriate comparison of the survival of implants placed with and without MSFA for meta-analysis or systematic review.

When studying the relationship between MBL and MSFA, it is necessary to discriminate MSFA from other types of bone grafting because the coronal implant contacts with the native bone and the apical implant area contacts with bone substitutes in MSFA, while it is generally reversed in other types of bone grafting procedures. Consequently, we refrained from referring to studies including bone grafting in the mandibular or anterior areas, and included only the studies comparing implants in the posterior maxilla with or without MSFA. There is also a controversy with regards to the relationship between MBL and MSFA. Implants with MSFA were reported to exhibit increased MBL than those in the native maxillary bone [15,30], but Sbordone et al. reported similar MBL measurements at the buccal aspect from implants in the MSFA area and the native bone in the posterior maxilla [10]. Meanwhile, implants in the sinus augmented group exhibited less bone resorption around them than those without sinus augmentation [29]. To determine the relationship between MBL and implants placed with and without MSFA in the maxillary molars, the present study used GEE analysis considering multiple implants within the same patient and the median value, 2.4 mm, which was the middle value equal to 50% in the measured MBL values from this study, as the reference. MBL values were prepared as a binary outcome variable (i.e., MBL < 2.4 mm or ≥ 2.4 mm). According to the multivariable analysis result of this study, MSFA was not statistically significant for the risk of MBL ≥ 2.4 mm although the OR decreased to 0.856. Only the location of the implant was significantly relevant to MBL ≥ 2.4 mm, as depicted in Table 5. Compared to the first maxillary molar site, the second maxillary molar site was about two-times more at risk for MBL ≥ 2.4 mm with statistical significance. The location of the implant has been mentioned to be associated with implant failure, and the survival rates of 6 mm implants were 100% in mandibular posterior implants and 87% for maxillary posterior implants [33]. In the comparison of short implants placed in the partial edentulous area of the maxilla and mandible with a 15-year follow-up, significantly higher MBL was observed in the maxilla than the mandible, while the implant position was not significantly influential on the implant survival [34]. In other retrospective studies, local factors, including the implant location, showed a more significant relationship with MBL than systemic factors [35], and the implants in the maxilla presented with significantly higher MBL than those in the mandible in a study with more than four-year follow-up that considered multiple implants in the same patient [36]. In the comparison of MBL around tilted and straight implants in the posterior maxilla, tilted implants replacing premolars showed significantly higher MBL than those for molars [37]. By way of explanation for these findings, the lower bone density of the maxilla than the mandible or discrepancy in the occlusal load in locations is usually considered plausible. Although prospective studies with more samples and additional analysis with other reference values are required, the results of this study showed that the second maxillary molar sites showed greater risk of more MBL of implants than the first maxillary molar site, irrespective of MSFA. Therefore, to prevent MBL in maxillary posterior implants, it is suggested that dentists pay more attention to surgical procedure and principles, occlusal adjustment, and maintenance therapy when treating the second molar than the first molar.

Based on the follow-up period, the possibility of implant survival increased more than 13 times in group 3 (15 ≤ years) compared to that of group 1 (5 ≤ years < 10) without statistical significance. For the estimate of MBL, the estimates were −1.84 and −2.32 for group 2 (10 ≤ years < 15) and group 3 (15 ≤ years) respectively compared to that of group 1 (5 ≤ years < 10) with significance. Smoking was generally reported to increase implant failure and MBL in the posterior maxilla [5,14,15]. The least successful patient in this study underwent the removal of all four implants placed with MSFA in maxillary molars. His implants located at the right maxillary molar sites were removed at the 8.87-year follow-up, and those at the left maxillary molar sites were removed at the 14.3-year follow-up. He was a heavy smoker with more than two packs of cigarettes per day, had controlled diabetes, and did not present with post-operative maxillary sinusitis. Smoking is suspected to be the major cause of implant failure for this patient, but smoking did not show any statistical significance for implant failure and MBL in this study.

Interestingly, postoperative maxillary sinusitis occurred in only three patients (five implants) although sinus membrane perforation happened in 25 patients (58 implants). Only one patient (two implants) had sinus membrane perforation out of three patients who had suffered from sinusitis after MSFA. Thus, it is suggested that maxillary sinus membrane perforation during MSFA may not always cause postoperative maxillary sinusitis if proper postoperative medication and care are accompanied after MSFA procedure. The strengths of the present study are that repeated observations within the same patients were considered to use the frailty model and GEE analysis, and that the genuine long-term effect of MSFA was evaluated by comparing implants placed in the maxillary molars with and without MSFA within the same host and operator factors. But these strict criteria restricted the number of patients and implants included. One of the limitations of this study is the small numbers of patients and implants. Second, although periapical radiographs with the paralleling technique were used, the MBL measurement from radiographs cannot reflect the buccal or palatal marginal bone resorption, which is more frequently encountered than mesial or distal bone resorption. Lastly, this study failed to reveal important confounding factors, such as parafunctional habits, masticatory force, opposing dentition, and compliance of periodic check-up due to the retrospective study.

## 5. Conclusions

Within the limits of this study, MSFA was significantly related with neither implant failure nor MBL after the 5.85- to 22-year follow-up when comparing implants placed in maxillary molars with and without MSFA within the same patients.

## Figures and Tables

**Table 1:** Characteristics of patients and implants placed in maxillary molars with and without maxillary sinus floor augmentation (MSFA).

Characteristics			
**Patient-related variables (No. of patients = 38)**			
Sex	Male	28 (73.7%)	
	Female	10 (26.3%)	
Age (years)	<50	19 (50%)	
	≥50	19 (50%)	
Smoking		21 (55.3%)	
Rhinitis		3 (7.9%)	
Diabetes		11 (28.9%)	
Osteoporosis		5 (13.2%)	
**Implant-related variables (No. of implants = 119)**			
		Native bone	MSFA
Implants placed in		24 (20.17%)	95 (79.83%)
Implant diameter (mm)	5.21 ± 0.53	5.17 ± 0.48	5.22 ± 0.55
Implant length (mm)	12.78 ± 1.28	12.48 ± 1.39	12.86 ± 1.25
Survived implants	106 (89.08%)	21	85
Failed implants	13 (10.92%)	3	10
Time of implantation			
immediately after extraction	14 (11.76%)	3 (12.5%)	11 (11.58%)
2 ≤ months after extraction < 4	7 (5.88%)	3 (12.5%)	4 (4.21%)
4 ≤ months after extraction	98 (82.35%)	18 (75.0%)	80 (84.21%)
Follow-up period (years)	12.62 ± 3.64	10.98 ± 3.12	13.04 ± 3.65
5 ≤ follow-up years < 10	32 (26.89%)	9 (37.50%)	23 (24.21%)
10 ≤ follow-up years < 15	54 (45.38%)	13 (54.17%)	41 (43.16%)
15 ≤ follow-up years	33 (27.73%)	2 (8.33%)	31 (32.63%)
Healing time (months)	7.70 ± 3.18	7.25 ± 2.95	7.82 ± 3.25
Marginal bone loss (mm)	3.15 ± 2.51	3.15 ± 2.61	3.15 ± 2.50
Residual bone height (mm)	5.27 ± 4.90	14.49 ± 1.71	2.94 ± 1.48
Perforation of sinus membrane during MSFA			
no		0 (0%)	37 (38.95%)
yes–diameter ≤ 5 mm		0 (0%)	44 (46.32%)
–diameter > 5 mm		0 (0%)	14 (14.74%)
Postoperative maxillary sinusitis			
no		0 (0%)	90 (94.74%)
yes		0 (0%)	5 (5.26%)
Location			
#16, 26	59 (49.58%)	13 (54.17%)	46 (48.42%)
#17, 27	60 (50.42%)	11 (45.83%)	49 (51.58%)

MSFA means maxillary sinus floor augmentation. Data are shown as numbers of patients, implants (%), or mean ± standard deviation. #, FDI World Dental Federation notation system.

**Table 2:** Univariable analysis using a frailty model for implant failure.

Variables			HR	95% CI	*p*-Value
Patient level	Sex	male (ref. female)	2.764	0.290–26.303	0.3764
	Age (years)	≥50 (ref. < 50)	0.478	0.112–2.043	0.3193
	Diabetes	yes (ref. no)	1.851	0.433–7.918	0.4062
	Osteoporosis	yes (ref. no)	0.832	0.075–9.193	0.8808
	Rhinitis	yes (ref. no)	1.837	0.146–23.038	0.6375
	Smoking	yes (ref. no)	1.249	0.282–5.528	0.7693
Implant level	MSFA (ref. native bone)		0.406	0.094–1.763	0.2290
	Time of implantation				
	Immediately after ext. (ref. 4 ≤ months after ext.)		Inf.	Inf.	0.9947
	2 ≤ months after ext. < 4 (ref. 4 ≤ months after ext.)		1.062	0.101–11.124	0.9601
	Diameter (mm)		0.997	0.300–3.319	0.9964
	Length (mm)		0.782	0.492–1.243	0.2982
	Healing time (months)		0.969	0.791–1.188	0.7625
	Residual bone height		1.038	0.914–1.179	0.5622
	Perforation of sinus membrane during MSFA (ref. no)		0.803	0.161–3.998	0.7885
		diameter ≤ 5 mm (ref. no)	0.841	0.153–4.639	0.8428
		diameter > 5 mm (ref. no)	0.688	0.050–9.442	0.7798
	Postoperative maxillary sinusitis (ref. no)		3.004	0.244–36.991	0.3906
	Location	#17, 27 (ref. #16, 26)	0.558	0.180–1.727	0.3116

HR, hazard ratio; 95% CI, 95% confidence interval; ref., reference; MSFA, maxillary sinus floor augmentation; ext., extraction; Inf, undetected value due to low sample number; Healing time, a period from implant placement to prostheses delivery; #, FDI World Dental Federation notation system.

**Table 3:** Multivariable analysis using a frailty model for implant failure.

Variables			HR	95% CI	*p*-Value
Implant level ^†^	MSFA (ref. native bone)		0.342	0.065–1.809	0.2069
	Time of implantation				
	Immediately after ext. (ref. 4 ≤ months after ext.)		Inf.	Inf.	0.9930
	2 ≤ months after ext. < 4 (ref. 4 ≤ months after ext.)		1.117	0.064–19.354	0.9396
	Diameter (mm)		0.784	0.176–3.486	0.7491
	Length (mm)		0.734	0.431–1.249	0.2537
	Healing time (months)		0.921	0.687–1.233	0.5799
	Residual bone height		1.057	0.920–1.214	0.4331
	Perforation of sinus membrane during MSFA (ref. no)		0.541	0.079–3.696	0.5308
		diameter ≤ 5 mm (ref. no)	0.606	0.081–4.541	0.6258
		diameter > 5 mm (ref. no)	0.295	0.010–8.509	0.4770
	Postoperative maxillary sinusitis (ref. no)		4.928	0.262–92.760	0.2868
	Location	#17, 27 (ref. #16, 26)	0.528	0.168–1.659	0.2740

^†^ adjusted by age at implant placement, sex, diabetes, osteoporosis, rhinitis, and smoking. HR, hazard ratio; 95% CI, 95% confidence interval; MSFA, maxillary sinus floor augmentation; ref., reference; ext., extraction; Inf, undetected value due to low sample number; healing time, a period from implant placement to prostheses delivery; #, FDI World Dental Federation notation system.

**Table 4:** Univariable analysis using generalized estimation equations (GEE) for marginal bone loss.

Variables			OR	95% CI	*p*-Value
Patient level	Sex	Male (ref. Female)	3.156	1.133–8.791	0.0279
	Age (years)	≥50 (ref. < 50)	0.579	0.214–1.566	0.2819
	Diabetes	yes (ref. no)	2.137	0.781–5.847	0.1394
	Osteoporosis	yes (ref. no)	0.634	0.226–1.779	0.3862
	Rhinitis	yes (ref. no)	2.442	0.289–20.631	0.4121
	Smoking	yes (ref. no)	1.281	0.483–3.400	0.6191
Implant level	MSFA (ref. native bone)		0.900	0.402–2.016	0.7972
	Time of implantation				
	Immediately after ext. (ref. 4 ≤ months after ext.)		1.772	0.574–5.466	0.3197
	2 ≤ months after ext. < 4 (ref. 4 ≤ months after ext.)		0.933	0.266–3.266	0.9132
	Diameter (mm)		1.649	0.765–3.553	0.2018
	Length (mm)		1.010	0.808–1.263	0.9275
	Healing time (months)		0.982	0.842–1.146	0.8201
	Residual bone height		0.996	0.933–1.063	0.8969
	Perforation of sinus membrane during MSFA (ref. no)		1.001	0.399–2.510	0.9987
		diameter ≤ 5 mm (ref. no)	0.952	0.353–2.570	0.9228
		diameter > 5 mm (ref. no)	1.180	0.417–3.340	0.7556
	Postoperative maxillary sinusitis (ref. no)		3.745	0.349–40.250	0.2758
	Location	#17, 27 (ref. #16, 26)	1.848	1.060–3.220	0.0303

OR, odds ratio; 95% CI, 95% confidence interval; ref., reference; MSFA, maxillary sinus floor augmentation; ext., extraction; healing time, a period from implant placement to prostheses delivery; #, FDI World Dental Federation notation system.

**Table 5:** Multivariable analysis using generalized estimation equations (GEE) for marginal bone loss.

Variables			OR	95% CI	*p*-Value
Implant level ^†^	MSFA (ref. native bone)		0.856	0.351–2.088	0.7325
	Time of implantation				
	Immediately after ext. (ref. 4 ≤ months after ext.)		1.430	0.473–4.324	0.5259
	2 ≤ months after ext. < 4 (ref. 4 ≤ months after ext.)		0.922	0.236–3.603	0.9074
	Diameter (mm)		1.604	0.680–3.781	0.2802
	Length (mm)		1.026	0.796–1.323	0.8428
	Healing time (months)		0.985	0.851–1.140	0.8365
	Residual bone height		1.000	0.931–1.073	0.9892
	Perforation of sinus membrane during MSFA (ref. no)		0.818	0.275–2.430	0.7171
		diameter ≤ 5 mm (ref. no)	0.780	0.246–2.471	0.6730
		diameter > 5 mm (ref. no)	0.971	0.275–3.430	0.9639
	Postoperative maxillary sinusitis (ref. no)		4.074	0.299–55.468	0.2917
	Location	#17, 27 (ref. #16, 26)	1.964	1.080–3.571	0.0270

^†^ adjusted by age at implant placement, sex, diabetes, osteoporosis, rhinitis, and smoking. OR, odds ratio; 95% CI, 95% confidence interval; MSFA, maxillary sinus floor augmentation; ref., reference; ext., extraction; healing time, a period from implant placement to prostheses delivery; #, FDI World Dental Federation notation system.

**Table 6:** Comparison of implant survival based on follow-up period (years).

Follow-up Period (Years)	Number of Implant		Simple GEE Model (Ref. Failure)				Multiple GEE Model (Ref. Failure) *			
	Survival	Failure	OR	95% CI		*p*-Value	OR	95% CI		*p*-Value
5 ≤ Y < 10	26	6	1.00	1.00	1.00	_	1.00	1.00	1.00	_
10 ≤ Y < 15	48	6	2.53	0.53	12.20	0.2461	2.15	0.28	16.29	0.4571
15 ≤ Y	32	1	17.22	0.37	790.92	0.1450	13.67	0.57	330.53	0.1076

Y, years; ref., reference; OR, odds ratio; 95% CI, 95% confidence interval; * adjusted by age at implant placement, sex, diabetes, osteoporosis, rhinitis, and smoking.

**Table 7:** Comparison of marginal bone loss based on follow-up period (years).

Follow-up Period (Years)	Marginal Bone Loss		Simple GEE Model				Multiple GEE Model *			
	N	Mean ± SD	E	95% CI		*p*-Value	E	95% CI		*p*-Value
5 ≤ Y < 10	32	3.92 ± 3.28	0.00	0.00	0.00	_	0.00	0.00	0.00	_
10 ≤ Y < 15	54	2.93 ± 2.31	−2.02	−3.79	−0.25	0.0250	−1.84	−3.71	0.03	0.0539
15 ≤ Y	33	2.77 ± 1.79	−2.56	−4.13	−0.99	0.0014	−2.32	−3.81	−0.83	0.0023

Y, years; SD, standard deviation; E, Estimate; 95% CI, 95% confidence interval; * adjusted by age at implant placement, sex, diabetes, osteoporosis, rhinitis, and smoking.

## Supplementary Materials

Table S1, Distribution of implant length, diameter, type, and manufacturer, is available online at https://www.mdpi.com/2077-0383/10/7/1360/s1.
