# Supplementary material for: Long-Term Comparison of Survival and Marginal Bone of Implants with and without Sinus Augmentation in Maxillary Molars within the Same Patients: A 5.8- to 22-Year Retrospective Study
Source: J Clin Med. 2021 Mar 25;10(7):1360. doi: 10.3390/jcm10071360 (PMC8036778; doi:10.3390/jcm10071360)
Supplement: Supplementary file 1 [file jcm-10-01360-s001.pdf]

Supplementary Table S1. Distribution of implant length, diameter, type, and manufacturer.

**Implant characteristic**		Native bone	MSFA	Sum
**Implant length (mm)**	8	1	0	1
	10	1	9	10
	11.5	1	1	2
	12	9	25	34
	13	6	20	26
	14	6	40	46
	Sum	24	95	119
Implant diameter (mm)	3.8	0	1	1
	4.0	1	4	5
	4.5	0	2	2
	5.0	18	60	78
	5.3	0	1	1
	6.0	5	27	32
	Sum	24	95	119
Implant manufacturer	Steri oss	22	91	113
	3i	0	2	2
	Lifecore	2	0	2
	IMZ	0	2	2
	Sum	24	95	119

MSFA, maxillary sinus floor augmentation.
